# Vascular Dysfunction in a Transgenic Model of Alzheimer's Disease: Effects of CB1R and CB2R Cannabinoid Agonists

**DOI:** 10.3389/fnins.2016.00422

**Published:** 2016-09-16

**Authors:** Jorge Navarro-Dorado, Nuria Villalba, Dolores Prieto, Begoña Brera, Ana M. Martín-Moreno, Teresa Tejerina, María L. de Ceballos

**Affiliations:** ^1^Department of Pharmacology, School of Medicine, Complutense University of MadridMadrid, Spain; ^2^Department of Physiology, Faculty of Pharmacy, Complutense University of MadridMadrid, Spain; ^3^Neurodegeneration Group, Cellular, Molecular and Developmental Neurobiology and CIBERNED, Cajal Institute, CSICMadrid, Spain

**Keywords:** Alzheimer's disease, β-amyloid, cannabinoid receptors, collagen IV, Tg APP, vascular dysfunction

## Abstract

There is evidence of altered vascular function, including cerebrovascular, in Alzheimer's disease (AD) and transgenic models of the disease. Indeed vasoconstrictor responses are increased, while vasodilation is reduced in both conditions. β-Amyloid (Aβ) appears to be responsible, at least in part, of alterations in vascular function. Cannabinoids, neuroprotective and anti-inflammatory agents, induce vasodilation both *in vivo* and *in vitro*. We have demonstrated a beneficial effect of cannabinoids in models of AD by preventing glial activation. In this work we have studied the effects of these compounds on vessel density in amyloid precursor protein (APP) transgenic mice, line 2576, and on altered vascular responses in aortae isolated ring. First we showed increased collagen IV positive vessels in AD brain compared to control subjects, with a similar increase in TgAPP mice, which was normalized by prolonged oral treatment with the CB1/CB2 mixed agonist WIN 55,212-2 (WIN) and the CB2 selective agonist JWH-133 (JWH). In Tg APP mice the vasoconstriction induced by phenylephrine and the thromboxane agonist U46619 was significantly increased, and no change in the vasodilation to acetylcholine (ACh) was observed. Tg APP displayed decreased vasodilation to both cannabinoid agonists, which were able to prevent decreased ACh relaxation in the presence of Aβ. In summary, we have confirmed and extended the existence of altered vascular responses in Tg APP mice. Moreover, our results suggest that treatment with cannabinoids may ameliorate the vascular responses in AD-type pathology.

## Introduction

Alzheimer's disease (AD) is the major cause of dementia. This neurologic condition is characterized pathologically by β-amyloid (Aβ) deposition, neurofibrillary tangles, composed of hypophosphorylated tau, the degeneration of particular subsets of neurons and neuroinflammation, as a consequence of glial activation. Although the existence of hereditary AD, with early onset, has been described, it only accounts for a small percentage of cases (Hardy, 1996; Campion et al., 1999). The actual cause of sporadic AD is unknown, but several risk factors have been recognized (Grammas, 2011; Carnevale et al., 2016; Hamel et al., 2016). Indeed, hypertension, hypercholesterolaemia, ischaemic stroke, the ApoE4 allele and diabetes, all characterized by a vascular pathology, constitute risk factors for AD. On the other hand, several abnormalities in cerebrovascular vessels have been observed, including amyloid cerebral angiopathy (Thomas et al., 2000; Hardy and Selkoe, 2002; Kalaria, 2002; Iadecola, 2010), with a prominent accumulation of Aβ in vessels, alterations in smooth muscle or endothelial cells, and thickening of basement membrane (Mancardi et al., 1980; Kalaria, 2002; Iadecola, 2010; Morris et al., 2014). Moreover, there are pathophysiological links among these actors, since increased hypertension in mice results in Aβ deposition and cognitive impairment (Carnevale et al., 2012). Similarly, in transgenic models of the disease exists angiopathy, alterations in cerebral microvasculature occur, with the presence of apoptotic vascular cells in brain (Christie et al., 2001; Miao et al., 2005; Tong et al., 2005).

Aβ induces several types of vessel dysfunctions. Indeed, preincubation of aortae rings with the peptide diminishes the vasodilator activity of acetylcholine (ACh), while the vasoconstrictor responses to phenylephrine (Thomas et al., 1996) and endothelin-1 (ET-1; Crawford et al., 1998) are enhanced. Free radical generation appeared to mediate the effects of Aβ, since the addition of the antioxidant enzyme superoxide dismutase (SOD) avoided Aβ effects (Thomas et al., 1996; Crawford et al., 1998). On the other hand, calcium channel blockers or calcium chelators fully abrogate the enhancement induced by Aβ on ET-1 vasoconstriction (Crawford et al., 1998). In regard to the chemical species, it has been reported that Aβ 1-40 appears to be the fragment inducing higher vasoactivity (Crawford et al., 1998; Smith et al., 2007), compared to fragments 1–42 or 25–35, both showing greater cytotoxicity. It should be noted that Aβ1-42, more prone to aggregation, along with the 1-40 peptide fragment are deposited in senile plaques. However, Aß1-40 is the chemical species present in blood. However, it is not clear whether the presence of endothelium is required for vasoactivity (Thomas et al., 1996; Crawford et al., 1998). Interestingly, Aβ intra-arterial infusion to rats decreased blood flow and increased vascular resistance specifically in cerebral cortex (Suo et al., 1998), and enhances mean arterial blood pressure (Arendash et al., 1999). In isolated middle cerebral arteries from amyloid precursor protein transgenic mice (Tg APP) the vasodilator responses to calcitonin gene related peptide (CGRP) and ACh were significantly reduced, although the vasoconstriction induced by ET-1 was preserved, and both catalase and SOD addition restored to control values ACh-induced vessel relaxation (Tong et al., 2005). Moreover, Tg APP mice showed selective impairment in endothelium-dependent regulation of the neocortical microcirculation, as measured by laser-Doppler, which was counteracted by SOD (Iadecola et al., 1999).

Cannabinoids are molecules interacting with cannabinoid receptors, or with similar chemical structure to tetrahydrocannabinol, the major constituent of *Cannabis sativa*. Thus, cannabinoid agonists comprise molecules derived from the plant, synthetic molecules, with higher potency, and the endocannabinoids, present in living animals. Anandamide (AEA) and 2-arachidonoylglycerol (2-AG) are the major endocannabinoids, which along with their synthetic and degrading enzymes, and specific cannabinoid receptors constitute the endocannabinoid system (Di Marzo and De Petrocellis, 2012; Pertwee, 2012), which has a modulatory role with pleiotropic actions. Cannabinoid agonists have shown neuroprotective and anti-inflammatory effects of interest for the treatment of different neurodegenerative (Baker et al., 2000; Glass et al., 2000; Arévalo-Martín et al., 2008; Fernández-Ruiz et al., 2011) and mental disorders (Marsicano et al., 2002; de Bitencourt et al., 2013; Leweke et al., 2016). We (Ramírez et al., 2005; Martín-Moreno et al., 2012) and others (Wu et al., 2013; Aso and Ferrer, 2014; Cheng et al., 2014) have described beneficial effects relevant for AD treatment. Indeed, cannabinoid agonists rescue the cognitive impairment in AD animal models, affording neuroprotection by decreasing neuroinflammation and Aβ levels. On the other hand, cannabinoid agonists are hypotensive agents. Their cardiovascular actions are complex (Randall et al., 2004; López-Miranda et al., 2008). They cause vasorelaxation of isolated vessels *in vitro*, and *in vivo* they induce multiphasic responses that lead to sustained hypotension. For instance, anandamide, the endocannabinoid, caused a triphasic response in anaesthesized rats: first, there is a hypotensive response, vagally mediated, followed by a pressor response and by a sustained hypotension (Varga et al., 1996). Moreover, WIN 55,212-2 and HU-210 in conscious rats induced pressor, and renal and mesenteric vasoconstrictor effects, but hindquarters vasodilator actions (Gardiner et al., 2001). Although in some instances the classical cannabinoid receptors, the well characterized CB_1_ and CB_2_ receptors, are involved in such responses, in other occasions different receptors are activated, the release of endothelial mediators may be implicated, or even direct effects on transduction mechanisms have been invoked.

Given the vascular alterations observed in AD and in its animal models, and that cannabinoid agonists show vascular effects, in this work we sought to investigate the vascular responses of two pharmacologically distinct cannabinoid agonists, the CB_1_/CB_2_ mixed agonist WIN 55,212-2 (WIN) and the CB_2_ selective agonist JWH-133 (JWH). We selected WIN because it shows a slightly higher CB_2_ selectivity compared to other mixed agonists, and JWH because it was one of the first CB_2_ selective agonists synthesized and characterized (Huffman et al., 1999). Since we have been using both compounds for years, commencing with our seminal work on the cannabinoid receptor alterations in AD and the effects of cannabinoid agonists on its experimental *in vitro* and *in vivo* models (Ramírez et al., 2005), we have gathered a broad knowledge on their pharmacology. Furthermore, we tested whether they counteract the Aβ-induced alteration in vessel function and if they maintain their effects in vessels of a transgenic mouse model of the disease, Tg APP mice (line 2576). The possible beneficial effects of cannabinoid agonists on the vascular system may be of therapeutic interest in a multifactorial disease such as AD.

## Materials and methods

### Materials

ßA_1−40_ (Polypeptide Group, France) was dissolved in PBS (1.72 mg/ml), aliquoted and stored at −80°C until used. WIN was purchased from Sigma, JWH was from Tocris (Cookson Ltd., UK), SR141716 (SR1; Rinaldi-Carmona et al., 1994) and SR144528 (SR2; Rinaldi-Carmona et al., 1998) were kindly donated by Sanofi-Synthelabo (Montpellier, France). For *in vitro* experiments each of these compounds was dissolved in DMSO at 10 mM, aliquoted and stored at −80°C. Before their use, drugs were diluted in appropriate solvent and DMSO never exceeded 0.1% in pharmacological experiments. For *in vivo* experiments, WIN and JWH were initially dissolved in chloroform (on ice), quickly aliquoted to prevent evaporation, dried under a stream of N_2_, and aliquots stored desiccated. Before their use, drugs were diluted in ethanol and added to the drinking water. Salts and other reagents were analytical grade from Merck.

### Human *post-mortem* brain tissue

For immunocytochemistry, cryoprotected and fixed frozen frontal cortex samples were obtained from the Neurologic Tissue Bank, Hospital Clinic, Barcelona, Spain, and processed as previously described (Ramírez et al., 2005). Human brains were obtained by the Neurologic Tissue Bank following written consent. Controls consisted of 3 males and 2 females (median 70.0, range 38.0–0.0 years of age; median 17.0, range 3.5–21.0 h of *post-mortem* interval), and clinically diagnosed and neuropathologically defined AD patients consisted of 3 females and 3 males (median 74.0, range 66.0–88.0 years; median 5.5, range 4.0–9.0 h).

### Animals and treatments

Tg APP transgenic mice were obtained via heterozygous breeding of mice expressing the 695 aa long isoform of the human APP containing a double mutation Lys 670-Asn, Met 671-Leu (swedish mutation) under transcriptional control of the hamster prion promoter on a C57BL/6 breeding background (Hsiao et al., 1996). Male Tg APP, and wild type (wt) littermates, used as controls, were 7 months old at the beginning of the experiments. Mice were group-housed (4–5 animals per cage) under controlled temperature (23 ± 2°C), with a 12:12 h light/dark cycle and with *ad libitum* access to food and water. All of the experiments were performed according to ethical regulations on the use and welfare of experimental animals of the European Union and the Spanish Ministry of Agriculture, and the procedures were approved by the bioethical committee of the CSIC.

WIN and JWH were administered in the drinking water at a dose of 0.2 mg/Kg/day using ethanol (0.1%) as vehicle (Martín-Moreno et al., 2012). The amount of water drank by the animals was assessed every other day and the treatment was adjusted to their weight. There was no difference in the body weight or the ingested water between groups, all along the experiment, discarding a possible reinforcing effect of cannabinoids.

Animals were sacrificed by cervical dislocation followed by decapitation at 11 months of age after 4 months chronic treatment. The brain was sagittally divided. One brain hemisphere was rapidly dissected on a cold plate, frozen on dry ice and stored at −80°C until assayed. The other hemisphere was immersion fixed in 4% paraformaldehyde (4% PF) in sodium phosphate buffer (PB) 0.1 M for 24 h, cryoprotected in sucrose 15% (24 h) and 30% (24 h) in PB, snap frozen in hexane (−60°C), and stored at −20°C until cut with a sliding microtome.

For pharmacological studies male mice, wt used as control, or Tg APP mice (line 2576) (25–30 g, 12 months of age) were sacrificed by decapitation following cervical dislocation. The thoracic aorta was removed, cleaned and cut into segments of 2 mm length. Rings were mounted in Multy Myograph System 610M (Danish Myo Technology, Denmark) at 37 ± 0.5°C and gassed continuously with a mixture of 95% O_2_-5% CO_2_, in a solution of the following composition: PSS (mM): NaCl 140, KCl 5, MgCl_2_ 1, CaCl_2_ 1.5, HEPES 5, and glucose 10. After equilibration, arterial rings were mounted between two parallel tungsten wires under a resting tension of 2 g. The isometric force was digitalized by Myodaq 2.01 program (Danish Myo Technology, Denmark) and displayed on a personal computer.

Arteries were preconstricted with 123.5 mM K^+^ (KPSS) for 4 min, washed, and then a) concentration-response curves for phenylephrine (Phe, 0.1-10 μM) and the thromboxane analog U46619 (0.01-0.1 μM) were performed; b) in a different set of experiments arterial rings were preconstricted with a submaximal concentration of U46619 (0.03 μM) for 15 min and then vasodilation to ACh 10 μM was assessed. After 2 washes vessels were incubated with Aβ (1 μM) for 15 min, and stimulated with U46619 (0.03 μM) for 15 min followed by relaxation with ACh 10 μM. To assess the vasodilatory effects of WIN and JWH concentration-response curves (1nM-10 μM) were performed in segments preconstricted with U46619. Concentration-response curves for WIN and JWH were performed in arterial segments treated with the selective CB_1_ or CB_2_ antagonists (SR1 and SR2), added 5 min before preconstriction with U46619. To investigate the effect of cannabinoid agonists on ACh-induced vasodilation, agonists were added to arterial rings at 0.5 μM after 15 min incubation with Aβ (1 μM). Given that the effect of Aβ is irreversible (Thomas et al., 1996) different arterial rings were used for each experiment. Tension was expressed as mN/mm artery length, or as a percentage of initial preconstriction (either with K^+^ or U46619). Indeed each ring was its own control, avoiding the variance between the responses of different rings from the same animal (decreased responsivity as the rings approached the abdominal aortic region).

### Immunohistochemistry

Immunostaining was performed on floating sections (30 μm thick) as described (Gómez Del Pulgar et al., 2002). Sections were incubated with the different antibodies overnight at 4°C. Dilutions of antibodies were as follow: polyclonal anti-CB1 (1:900, CC2, raised in our laboratory, De March et al., 2008), polyclonal anti-collagen IV (Col IV; 1: 400, ref. CR013X; Fitzgerald, MA, USA). The CC2 antibody was raised in rabbits using as immunogen the 15 aa N-terminal end of the CB_1_ receptor protein coupled to keyhole limpet hemocyanin. The antiserum was affinity purified, and it was characterized in wt mice and in CB_1_ KO mice brain. CB_1_ immunoreactivity brain distribution was in agreement with previous studies (Tsou et al., 1997, 1999). The anti-collagen antibody has been raised in rabbits using as immunogen Col IV from human and bovine placenta. It shows negligible cross reaction with Col I, Col II, Col III, and Col V. Development was conducted by the Avidin-Biotin Complex (ABC) method (Pierce), and immunoreactivity was visualized by 3,3′-diaminobenzidine oxidation as chromogen, with (CB_1_) or without nickel enhancement (Col IV). Omission of primary or secondary antibodies resulted in no immunostaining.

Images were acquired with a Zeiss Axiocam high resolution digital color camera, using the same settings and segmentation parameters (MCID software; InterFocus Imaging, UK) for a given marker and experiment. The mean value for each animal per region results from the analysis of 5–6 sections. The percentage of the brain area covered by Col IV positive vessels was assessed by image analysis with MCID software.

### Analysis of mRNA levels by RT-PCR

Total RNA from pooled aortae (*n* = 3–4) was extracted using TRIzol reagent according to the manufacturer's instructions (Invitrogen). To avoid interference with potential genomic DNA amplification 1 μg of total RNA was treated with 1 μl DNAse I (Invitrogen) plus 1 μl of 10X Buffer (Invitrogen) and incubated for 15 min at RT, then EDTA (25 mM) was added and incubated at 65°C for 15 min to inactivate DNAse I. For cDNA synthesis a total of 1 μg of RNA were reverse-transcribed for 75 min at 42°C using 5 U of avian myeloblastosis virus reverse transcriptase (Promega) in the presence of 20 U of RNAsin (Promega). The PCR reaction was performed using TaQ polymerase (TaQ DNA polymerase Sigma) and a mixture of reverse and forward primers (5 pmol). The primers used were CB_1_ forward 5′-AGCTTTGTTGACTTCCAGTGT and CB_1_ reverse 5′-CTGCCCACAGATGCTGTGAA, CB_2_ forward 5′-AGGAGCTGTCAGCTCAGGGTAT and CB_2_ reverse 5′-CTGCGCCCCTAAGGACCTA. The PCR reaction (final volume10 μl) was performed in a Veriti thermal cycler (Applied Biosystems) and the PCR program was as follows: initial denaturation for 10 min at 95°C, then 40 cycles of denaturation (15 s, 95°C), annealing (30 s, 60°C), and extension (30 s, 60°C). The PCR products were analyzed by standard agarose gel electrophoresis, and gene expression levels were detected by the use of ethidium bromide.

### Electron microscopy

Aortae 1–2 mm long rings were fixed in 4% PF/ 2.5% glutaraldehyde in cold 0.1 M Na cacodylate buffer immediately after dissection, for 6 h. The segments were washed five times with cacodylate buffer every 30 min, and left overnight at 4°C. The segments were postfixed with 1% osmium tetroxide and potassium ferrocyanide in distilled water for 1.5 h, they were washed with distilled water (3 × 10 min washes) and dehydrated in increasing acetone solutions (50–100% each for 15 min). The segments were then gradually embedded in resin (1:3, 1:1, 3:1 acetone:pure resin) and finally left in pure resin (TAAB 812 mix) at 60°C overnight. The resin embedded samples were sectioned by diamond knife, and the 80 nm sections were collected onto copper grids and post-stained with 1% uranyl acetate and Reynolds lead citrate for 4 and 3 min, respectively. Electron micrographs were obtained using a Jeol JEM-1010 high resolution transmission electronic microscope (Jeol, Tokyo, Japan).

### Statistical analysis

In pharmacological experiments we used one vessel per mouse, therefore n represents number of animals. In brief, each aorta was cut into 5 different rings and each ring was used for a given treatment to avoid artefactual results. Results are expressed as mean ± standard error mean (SEM) or as mean ± standard deviation (SD). Statistical analysis was assessed by using two-way or one-way analysis of variance (ANOVA) followed by Wilcoxon's test, if the data follow a Gaussian distribution (KS normality test), or by Kruskal-Wallis test, followed by Dunn's test (version 5.0, Prism software, GraphPad, USA). A value of *p* < 0.05 was considered significant.

## Results

### Vascular density is increased in AD frontal cortex and Tg APP mice

**Figure 1:**
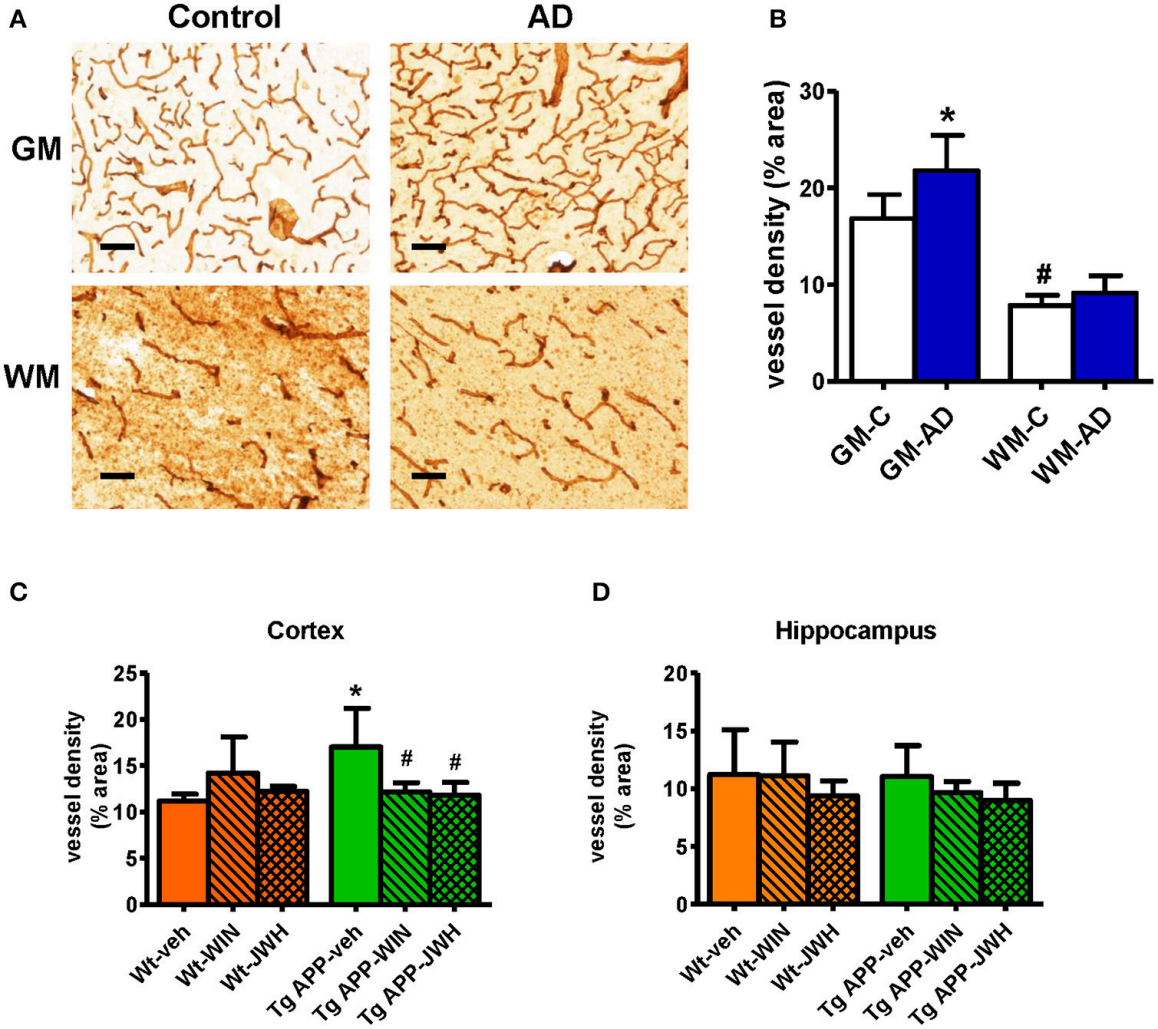
**Vessel density is altered in AD compared to controls. (A)** Representative collagen IV immunostaining of cortical vessels in human controls (*n* = 5) and AD patients (*n* = 6). Scale bar, 100 μm. GM, gray matter; WM, white matter. **(B)** Collagen IV positive vessels are significantly increased in gray matter. Results are mean ± SD (*n* = 5–6) and they are expressed as percentage of area occupied by vessels. ^*^p < 0.05 versus controls, ^#^*p* < 0.05 versus gray matter vessel density (Student's *t*-test). **(C)** Tg APP vehicle treated mice showed increased collagen IV vessel density in cortex vs. wild type (Wt) vehicle treated mice. Cannabinoid agonists normalized vessel density of Tg APP mice. Results are mean ± SD (*n* = 7–8) ^*^p < 0.05 vs. controls (Wt-veh), ^#^*p* < 0.05 vs. Tg APP-veh (Kruskal-Wallis, followed by Dunn's test). **(D)** No changes in vessel density were found in hippocampus due to genotype and/or drug treatment.

Previous studies have reported vascular alterations in the brain of AD affected individuals, such as increased vessel density and greater collagen deposition at the structural level. As shown in Figures 1A,B) we found an increase (≈30%) in Col IV positive vessel density in the gray matter of frontal cortex from AD patients compared with control subjects. Vessel density was significantly lower in the white matter compared to the gray matter. No difference in vessel density in the white matter was found between the control and AD group (Figures 1A,B). Vessel density in Tg APP mice was much higher (≈50%) in cortical areas compared to wt mice, however it showed similar density in the hippocampus (Figures 1C,D). Interestingly, prolonged *in vivo* oral treatment (0.2 mg/Kg/day) with both WIN, a mixed CB_1_/CB_2_ agonist, and JWH, a CB_2_ selective agonist, counteracted the increased Col IV vessel density. In summary, similar vessel alterations were found in the neurologic condition and in the experimental model of AD, where a prolonged oral treatment of a cannabinoid agonist prevented vascular changes.

### Vascular dysfunction in Tg APP mice, contribution of β-amyloid and vasodilatory effect of cannabinoid agonists

**Figure 2:**
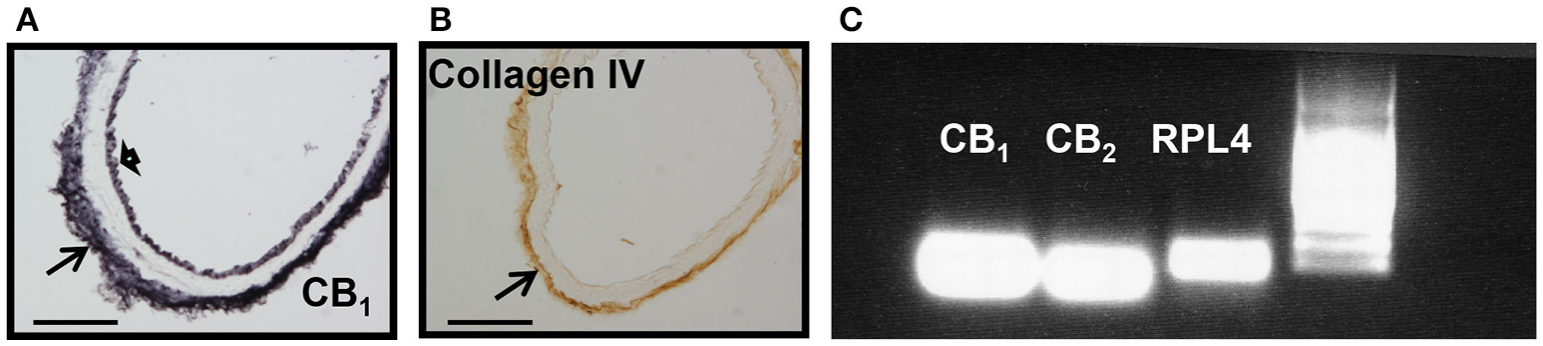
**CB_**1**_, CB_**2**_, and collagen IV expression in mouse aorta. (A)** CB_1_ immunostaining shown at endothelial cells (short arrow) and *basal lamina* (arrow), **(B)** while collagen IV is restricted to the *basal lamina* (arrow). Scale bar, 50 μm; representative images of *n* = 3 aortae for each immunostaining. **(C)** CB_1_ and CB_2_ mRNA expression in extracts from mouse aorta. RPL4 was used as control. Representative image of *n* = 3 independent experiments done with 3–4 pooled aortae.

We next examined whether mice aortae expressed CB_1_ and CB_2_ receptors. CB_1_ immunoreactivity has been previously reported in brain vessels (Ashton et al., 200), but the presence of CB_2_ receptors is uncertain. CB_1_ receptors were expressed in endothelial cells, at the *basal lamina*, but not in smooth muscle cells (Figure 2A, representative image of *n* = 3), while Col IV immunoreactivity just stained the *basal lamina* of the aorta (Figure 2B, representative image of *n* = 3). The immunostaining was very reproducible for both antibodies. There is debate on the specificity of CB_2_ receptors antibody (Cécyre et al., 2014; Li and Kim, 2015), therefore we used PCR to demonstrate CB_2_ and CB_1_ receptor expression in aorta extracts (Figure 2C).

**Figure 3:**
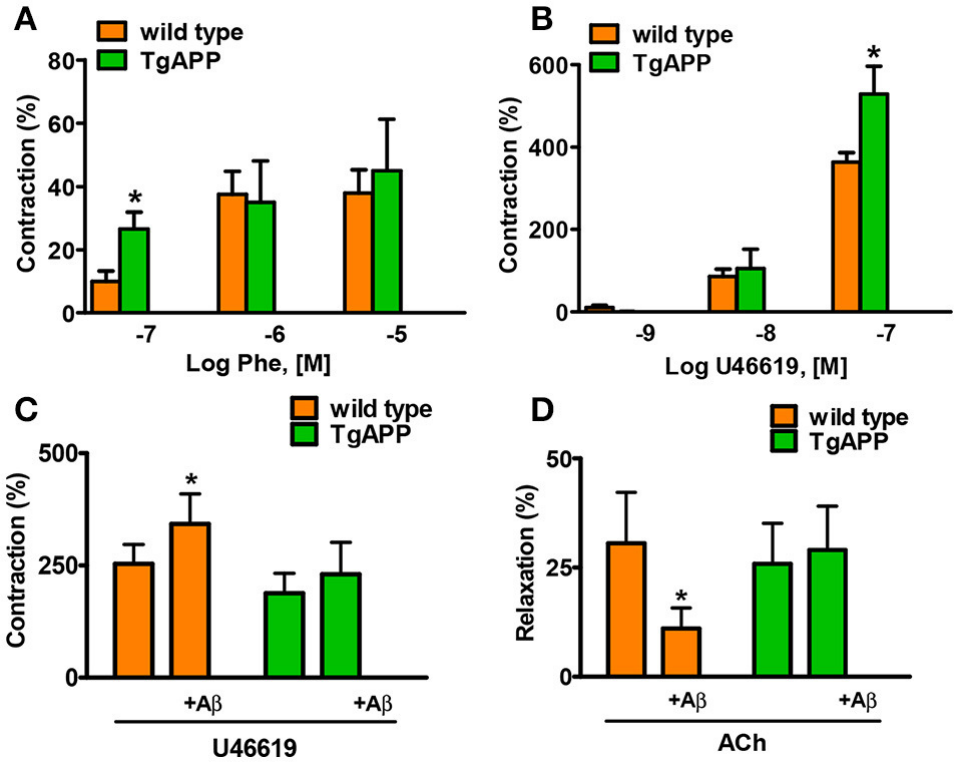
**Alterations in aorta contractility of Tg APP mice**. Tg APP aortae showed increased phenylephrine **(A)** and U46619-induced **(B)** contraction compared to aortae from wt mice. Vessels were precontracted by 123.5 mM K^+^ and washed before other treatment addition. Aβ (1 μM) incubation increased U46619 (0.03 μM) contraction **(C)** and decreased ACh (10 μM) relaxation **(D)** in wt aortae, but not in Tg APP aortae. Preconstriction levels were similar for all the vessels (approximately 5.44 mN). Results are mean ± SEM of *n* = 7 mice and are expressed as percentage of 123.5 mM K^+^-induced contraction, considered 100%. ^*^p < 0.05 (Kruskal-Wallis, followed by Dunn's test).

Constriction of aorta rings with high potassium (123.5 mM K^+^) was decreased by 50% in Tg APP mice aortae compared to those from wt mice (1.07 ± 0.13 and 2.25 ± 0.30 mN/mm respectively; *p* < 0.01, Student's *t*-test). Next, we tested two pharmacologically distinct vasoconstrictors: phenylephrine and the thromboxane analog U46619 (Figure 3). The vasoconstrictor response to 0.1 μM phenylephrine was enhanced by 2 fold in Tg APP compared to wt mice (Figure 3A), and that of 0.1 μM U46619 around a 50% (Figure 3B). We did not find any differences in the vasodilation induced by ACh (100 μM) between groups (data not shown). Furthermore, the cannabinoid agonists (15 min preincubation) under study did not change ACh vasodilation either (data not shown).

**Figure 4:**
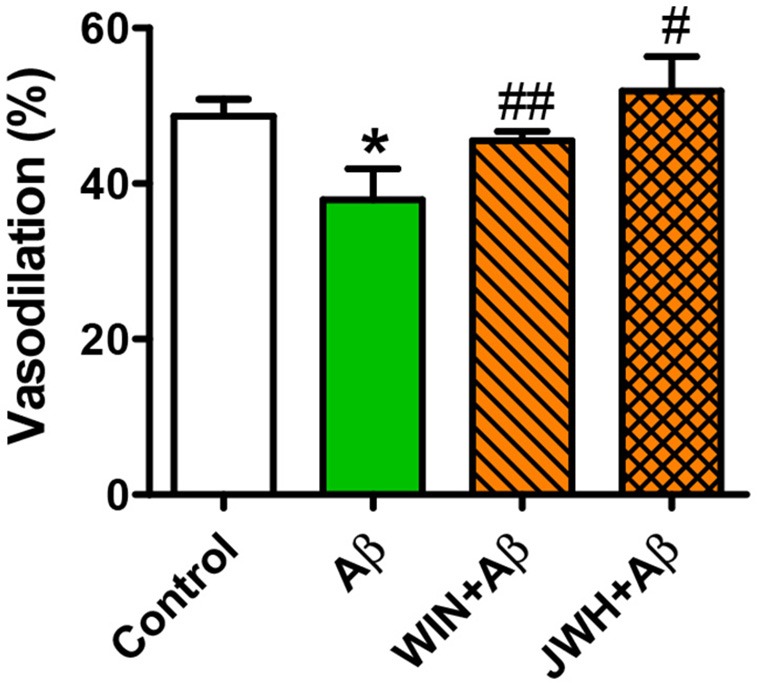
**Cannabinoid agonists prevent Aβ reduction of ACh relaxation**. Vessels from wt mice were precontracted with U46619 (0.03 μM) and ACh (10 μM) vasodilation assessed in the absence and presence of WIN and JWH. Preconstriction levels were similar for all the vessels (approximately 5.44 mN). Results are mean ± SEM of *n* = 6 mice and are expressed as percentage of U46619 contraction. ^*^p < 0.05 vs. untreated-aortae; ^#^*p* < 0.05; ^*##*^*p* < 0.01 vs. Aβ treated alone (Kruskal-Wallis, followed by Dunn's test).

Given that Tg APP mice are continuously exposed to circulating Aβ we wondered whether the peptide would mediate those responses. Aβ (1 μM) preincubation increased vasoconstriction to 0.03 μM U46619 (Figure 3C) and decreased vasodilation to 10 μM ACh (Figure 3D), in aortae from wt mice, although the peptide alone did not show any vasoactivity. However, incubation with Aβ did not alter arterial vasoconstriction or vasodilation in Tg APP mice aortae (Figures 3C,D). Interestingly, both cannabinoid agonists rescued ACh-induced vasodilation in the presence of Aβ (Figure 4) in wt mice.

**Figure 5:**
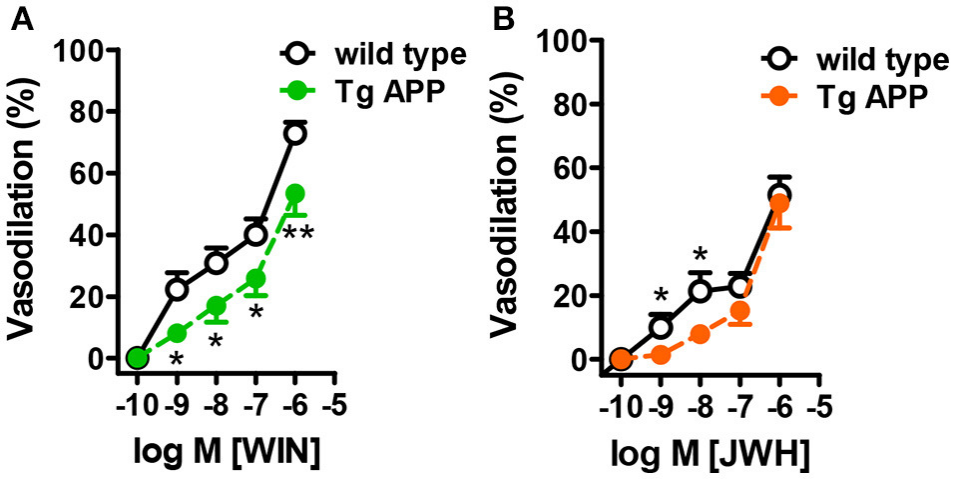
**Cannabinoid agonists induce dose-dependent vasodilation**. Cannabinoid vasodilation was reduced in Tg APP aortae compared to wt **(A,B)**. Concentration-response curves (1 nM-10 μM) for WIN and JWH were performed in segments preconstricted with U46619 (0.03 μM). Preconstriction levels were similar for all the vessels (approximately 8.9 mN). Results are mean ± SEM of *n* = 6 mice and are expressed as percentage of U46619-induced contraction. ^*^p < 0.05, ^**^p < 0.01 (two way ANOVA).

WIN concentration-dependently induced vasodilation in control mice, with a maximal effect of 80% at 1 μM (Figure 5A). The vasodilatory effect of JWH was smaller than the one induced by WIN, with a maximal effect of 56% at 1 μM (Figure 5B). In Tg APP aortae the vasodilation induced by WIN was significantly decreased at all the concentrations tested (Figure 5A), but in the case of JWH the effect at lower concentrations (1 and 10 nM) was decreased and at higher concentrations was similar between wt and Tg APP mice (Figure 5B).

Taken together these results show that vascular function is markedly altered in Tg APP mice and that Aβ may play a role in those altered responses. Furthermore, cannabinoid agonists induce vasodilation in aortic rings, which is partially preserved in Tg APP mice.

### Ultrastructural changes in Tg APP aortae

**Figure 6:**
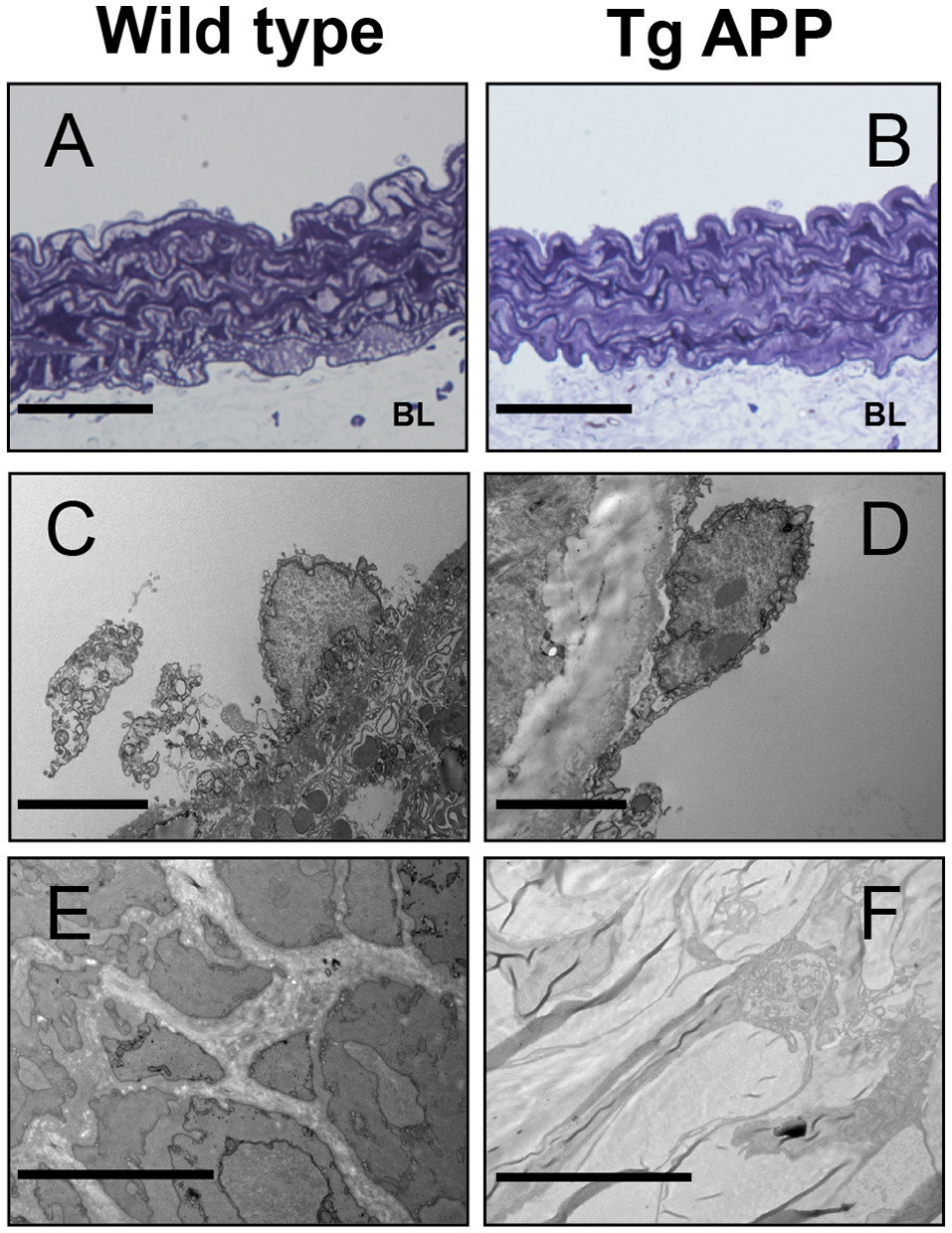
**Electron micrographs of wild type and Tg APP mice**. Representative images are shown for wt **(A,C,E)** and Tg APP mice aortae **(B,D,F)**. **(A,B)** toluidine blue micrographs showing similar structure (*n* = 20 animals/per group). BL: *basal lamina*, **(C,D)** endothelial cells show similar appearance (wt *n* = 4, Tg APP *n* = 6). **(E)** smooth muscle cells surrounded by collagen in wt mice aorta, **(F)** muscle cells are embedded in collagen in Tg APP aorta (wt *n* = 4, Tg APP *n* = 6). Scale bars, 50 μm in (**A,B**); 100 nm in **(C–F)**.

Some reports have described changes in the structure of Tg APP vessels (Christie et al., 2001; Tong et al., 2005). Therefore, we sought to determine if changes at the ultrastructural level may explain the vessel dysfunction observed in Tg APP mice. Toluidine labeled vessels showed similar vessel structure (Figures 6A,B). Endothelial cells appeared unaltered in both strains (Figures 6C,D). Moreover, smooth muscle cells also appeared normal, with normal numbers of mitochondria (not shown). However, there was a great difference in *basal lamina* collagen that was markedly increased in Tg APP when compared with wt aortae (Figures 6E,F).

## Discussion

In the present work we report that WIN, a mixed CB_1_ and CB_2_ agonist (Howlett et al., 2002), and JWH, a CB_2_ selective agonist (Huffman et al., 1999), induce vasodilation of isolated aortae. Tg APP vessels show altered vascular responses, in which Aβ may play a role, that were restored by the cannabinoids under study. We found an enhancement of collagen in *basal lamina*, that may partly explain the vascular dysfunction in Tg APP mice. This collagen increase was found in AD cerebrocortical vessels, and in Tg APP mice as well, and was fully reverted by prolonged oral treatment with both cannabinoid agonists. Taken together these results suggest that cannabinoid have effects on vascular function that may be beneficial in the treatment of AD.

Vessel function is compromised in Tg APP mice. Indeed we have confirmed and extended previous reports on the increase in the vasoconstriction to phenylephrine in isolated aorta rings (Thomas et al., 1996), and we have found similar increases with U46619, that decreased cerebral blood flow *in vivo* (Iadecola et al., 1999). However, endothelium-independent vasoconstriction was markedly reduced in Tg APP aortae, as judged by the decreased vasoconstriction to high potassium. This change parallels the attenuation in the vasodilator response to sodium nitroprusside (an endothelial-independent vasodilator) observed *in vivo* by multiphoton microscopy in Tg APP mice (Christie et al., 2001). Although ACh vasodilation was decreased in cerebral arteries from Tg APP mice (Tong et al., 2005), and following topical application onto the brain (Christie et al., 2001), in our hands its vasodilatory response was similar in wt and in Tg APP aortae. These results may be explained by the different origin of the vessels, cerebral compared to peripheral vessels, or the age of the animals. In AD, vessels are continuously exposed to high circulating levels of soluble Aβ, in contrast to the insoluble form of the peptide present in senile plaques occurring in brain. In our hands incubation with Aβ up to 15 min did not alter mice vessel tone. This is in contrast with the results of Thomas et al. (1996) and Crawford et al. (1998) obtained in rat aorta. Given that the methods used were very similar, we speculate that the rodent species accounts for this difference. However, in wt mice Aβ significantly enhanced the vasoconstriction to the thromboxane analog, paralleling the results obtained with noradrenaline, phenylephrine or ET-1 reported by other authors (Thomas et al., 1996; Crawford et al., 1998; Smith et al., 2007). Similarly, in the present study the vasodilation to ACh was decreased by Aβ (Smith et al., 2007) in wt mice. In contrast, the vessel responses in Tg APP were not modified by Aβ. These results suggest that in Tg APP mice, that express high levels of APP in the brain and in peripheral organs, including cerebral microvessels and the aorta (Paris et al., 2004), there is tolerance to Aβ effects due to the continuous exposure to the peptide. More importantly, both cannabinoids were able to normalize the Tg APP dysfunctional responses.

Cannabinoids induce vasodilatory effects in different isolated vessels, but so far these responses have not been studied in Tg APP mice. The CB_1_/CB_2_ mixed agonist WIN induced a concentration-dependent vasodilation of wt mice aortae, reaching 80% decrease of the maximal constriction to U46619, and higher than the vasodilation to ACh at 10 μM. The maximal vasodilatory effect to JWH in wt aortae was smaller compared to WIN. Cannabinoid-induced vasodilation, in spite of the presence of both CB_1_ (Ashton et al., 2004) and CB_2_ receptors in aortae, was completely insensitive to either CB_1_ or CB_2_ antagonism (data not shown). This is not without precedent, since the vascular effects of cannabinoids in many instances have been shown to be resistant to antagonism by cannabinoid antagonists, and they may involve activation of other targets (Randall et al., 2004; López-Miranda et al., 2008). We did not intend to characterize the mechanism underlying the vasodilatory effects of WIN and JWH in this work, since the pharmacology of the effects of cannabinoids is increasingly complicated (Randall et al., 2004; Stanley and O'Sullivan, 2014). Several possible targets could be proposed such as the putative “endothelial” cannabinoid receptor, potassium channel activation and calcium channel blockade. On the other hand, several cannabinoid agonists, including WIN, interact with peroxisome proliferator-activated receptors (PPAR) (O'Sullivan, 2016), members of the family of nuclear receptors, exerting vasodilation (O'Sullivan, 2007). Importantly, the vasodilation to both WIN and JWH was partially preserved in Tg APP mice, suggesting its possible therapeutic endorsement in AD.

We observed increased Col IV vessel density in AD specimens compared to control subjects, with a similar increase in Tg APP brain. Previous works have reported increased thickening of basement membranes in AD (Mancardi et al., 1980; Kalaria, 2002; Miao et al., 2005), in particular Col IV (Miao et al., 2005; Tong et al., 2005), associated or not with differences in density. In Tg APP mice similar changes were observed (Tong et al., 2005). Although the exact cause of increased basement membrane is unknown, several factors could be involved such as soluble Aβ and its progressive deposition in vessels, inflammatory mediators derived from activated glial cells around vessels and chronic changes in levels of vasoactive mediators (Grammas, 2011). Cannabinoid agonists, in particular CB_2_ selective agonists, impinge on several of these factors by decreasing glial activation, inflammation and Aβ levels (Ramírez et al., 2005; Martín-Moreno et al., 2012; Wu et al., 2013; Chiurchiù et al., 2015), explaining the normalization in vessel density following prolonged oral treatment with the drugs. At the ultrastructural level, aortic endothelial cells appeared normal in Tg APP aortae, in agreement with their preservation found in other works (Iadecola et al., 1999; Miao et al., 2005), which contrasts with the endothelial disruption in Aβ treated vessels (Thomas et al., 1996). Therefore, altered vessel function is not a consequence of endothelial disruption or death. Interestingly the major change observed in Tg APP aortae compared to wt mice was the increase in Col IV in the basement membrane, paralleling the changes in AD brain microvasculature, which may be involved in altered vessel contractility.

We have here described important pharmacological effects of cannabinoid agonists with relevance for the therapy of a devastating disorder such as AD. Prolonged oral treatment abrogated the changes in microvasculature that are important for vascular function and the perivascular drainage of Aβ from the parenchyma, that would initiate or worsen Aβ angiopathy, leading to a vicious circle toward further accumulation of the peptide. Moreover, both cannabinoids improved endothelial-dependent relaxations impaired by Aβ and showed vasodilatory effects that are maintained in Tg APP mice, albeit being reduced. Finally, the therapeutic activation of CB_2_R is safe and it does not trigger psychoactivity (Atwood and Mackie, 2010; Pertwee, 2012).

## Author contributions

MLC conceived the work. MLC, TT, and DP designed the study. JN-D, NV, BB, AM, and MLC performed the experiments and analyzed the data. MLC, JN-D, and NV wrote the article. All authors revised and approved the version to be published.

### Conflict of interest statement

The authors declare that the research was conducted in the absence of any commercial or financial relationships that could be construed as a potential conflict of interest.
